# Risk Factors Associated with the Prevalence of Antibodies Against Brucellosis in Equids from Western Pará, Brazil

**DOI:** 10.3390/microorganisms13071525

**Published:** 2025-06-30

**Authors:** Eloine Maria Bandeira Picanço, Francisco Flávio Vieira de Assis, Poliana Leão Peleja, Ana Beatriz Barbosa de Sousa, Raimundo Alves Barrêto Júnior, Ronaldo Francisco de Lima, Kedson Alessandri Lobo Neves, Antonio Humberto Hamad Minervino

**Affiliations:** 1 Laboratory of Animal Health, LARSANA, Federal University of Western Pará, UFOPA, Av. Vera Paz, S/N, Salé, Santarém 68040-255, PA, Brazil; eloine_picanco@hotmail.com (E.M.B.P.); ff.assis@outlook.com (F.F.V.d.A.); polianalepeleja5@gmail.com (P.L.P.); anabeatriz4t@gmail.com (A.B.B.d.S.); 2 Graduate Program in Society, Nature and Development, Federal University of Western Pará, UFOPA, Av. Vera Paz, S/N, Salé, Santarém 68040-255, PA, Brazil; 3 Department of Animal Science, Federal Rural University of the Semiarid Region, UFERSA, Mossoró 59625-900, RN, Brazil; barreto@ufersa.edu.br; 4 Instituto of Biodiversity and Forrest, IBEF, Federal University of Western Pará, UFOPA, Av. Vera Paz, S/N, Salé, Santarém 68040-255, PA, Brazil; ronaldofranciscolima@yahoo.com.br (R.F.d.L.); kedson_neves@hotmail.com (K.A.L.N.)

**Keywords:** equine brucellosis, BAPA, 2-ME, zoonosis, public health

## Abstract

This study aimed to determine the prevalence of antibodies against equine brucellosis in three distinct equid groups (farm animal, urban carthorse, and sport horse) in Western Pará, Brazil, and to identify associated risk factors. A cross-sectional study, involving 75 farms across 14 municipalities, sampled 1069 equids composed of urban carthorses and sport horses. Serum samples were tested for antibodies against *Brucella* spp. using the buffered acidified plate antigen (BAPA) test for screening, followed by the serum agglutination in tubes with 2-mercaptoethanol (2-ME) for confirmation. Univariate and multivariate analyses assessed associations between brucellosis and potential risk factors. Out of the 1059 equids sampled, 4.05% (95% CI: 3–5.5%) tested positive in the BAPA test. Of the 44 BAPA-positive samples, 11 were confirmed positive by 2-ME, 15 were inconclusive, and 18 were negative, yielding an animal-level prevalence of 1.03% (95% CI: 0.5–1.8%) for equine brucellosis in western Pará. Prevalence was higher in the Southwest Pará Mesoregion (*p* = 0.047) compared to the Lower Amazon Mesoregion. No significant associations were found with animal type, reproductive problems, animal sex, species, breed, and age group. Out of the 75 farms, 8 (10.66%) had at least one seropositive equid. Confirmed cases were identified in five municipalities (Brasil Novo, Uruará, Altamira, Rurópolis, and Itaituba). This first report of equine brucellosis in Western Pará underscores the need for enhanced surveillance and control measures to mitigate its zoonotic risk to humans and transmission to other animals.

## 1. Introduction

The state of Pará has one of the largest equine populations in Brazil, with more than 800,000 animals. Most of these animals are found on farms with livestock activity and are commonly used in the management of cattle and buffaloes and as a means of transport [1]. In addition, in the urban areas of Pará, equids are often used to transport materials and in sports such as *Vaquejada*, hoop competitions, drum, and horseback riding [2].

Infectious diseases in equids, besides the impact on animal health, may harm animals´ owners and pose a public health risk if the pathogen has zoonotic potential.. Among these diseases is equine brucellosis, a chronic, infectious-contagious, zoonotic disease caused by bacteria of the genus *Brucella* spp., which affects domestic mammals and some wild mammals [3]. Natural infection in horses happens directly or indirectly, mainly through the ingestion of water or food contaminated by infected material from the reproductive tract of cattle and pigs [4].

In Brazil, equids can be infected with *Brucella abortus*, *Brucella suis*, and *Brucella canis*; however, the most commonly described disease is *Brucella abortus*, due to the frequent cohabitation of these animals with cattle. In these animals, the clinical manifestations mainly result in reduced work capacity [5]. One of the most critical risk factors for equine brucellosis is the close contact with other animals susceptible to *Brucella* spp., particularly bovids, due to shared habitats or stables [6]. In addition, infected equids can be a potential source of infection for other species, including humans [3].

As a mechanism to control brucellosis, the National Program for the Control and Eradication of Bovine and Buffalo Brucellosis and Tuberculosis (PNCEBT) was introduced. The PNCEBT includes diagnostic testing (e.g., BAPA and 2-ME), mandatory euthanasia of positive animals, and vaccination of cattle (but not equids) to control brucellosis spread [7]. The same strategies of the program are used to control equine brucellosis.

Currently, the global prevalence of equine brucellosis is 1.92%, with the lowest and highest seroprevalence identified in Europe (0%) and Asia (10%), respectively [8]. In Brazil, there are few studies that investigate equine brucellosis, which limits the information about the disease distribution in the country. Isolated research in different states shows the presence of *Brucella* spp. antibodies in equids that may cause economic and public health losses [9,10].

Previous studies in Pará showed equine brucellosis prevalence ranging from 5.76% [11], to 1.03% [12] in studies on different regions then Western Pará (with distances ≥ 500 km). This study addresses this gap by providing the first epidemiological data on equine brucellosis in this region, critical for informing regional veterinary and public health policies [11].

In view of the severity of the disease, the importance of laboratory diagnosis for the identification of animals testing positive for brucellosis is evident. Because it is an incurable zoonotic disease, it can become an obstacle to the development of equideoculture [3,8]. Thus, this study aimed to evaluate the prevalence of antibodies against *Brucella* spp. in equids from Western Pará, and identifying associated risk factors, providing the first epidemiological data for this region.

## 2. Materials and Methods

### 2.1. Study Area

The study was conducted in Western Pará, north of Brazil, in the Amazon region. This region comprises 24 municipalities, of which 22 were included in the target population, totaling 51,231 equids distributed across 10,031 farms according to IBGE [1]. The climate of the region is of the Am type, according to the Köppen classification, with an average annual temperature of 25.9 °C and an average annual rainfall of 2150 mm, with a strong concentration between January and June and, more rarely, from July to December [13].

### 2.2. Study in Farm Animal

A cross-sectional study with one-stage cluster sampling was designed to estimate the seroprevalence of antibodies against brucellosis in horses raised on farms, considering a cluster a farm with horses according previously described work [2]. The number of clusters (Nc) was obtained according to the formula described by Thrusfield [14] considering the average number of equids per cluster, and the assumed prevalence.

To distribute the farms (clusters) among the 22 municipalities, stratified proportional sampling was performed, which considers the total number of farms in each municipality according to the formula: SN = (n × 100/N) × NT/100; where SN = sample number of farms in each municipality; n = number of farms in each municipality; N = total number of farms in the region; NT = sample size (previously calculated) = 101. Among the 22 municipalities, 18 with one or more farms were visited. From this previous study, samples from 14 municipalities in Western Pará were included in the present study.

The number of samples from each property was defined as the number of animals present at the time of the visit, according to the availability of the owners. A list with the name of the farmer, location of the farm, and number of horses, made available by the Agricultural Defense Agency of the State of Pará, was used to select the farms in each municipality.

### 2.3. Study in Sport Horses and Urban Carthorses

Horses raised for sports circulate between the municipalities to participate in events. Equids used as traction force in urban areas to transport goods (mainly ferrous waste and debris) are present in Brazil, known as urban carthorses. Considering that there was no information on the number of horses used for these purposes, convenience sampling was performed [15].

### 2.4. Sample Collection

The samples used in this study came from the analyses of the following studies: Minervino et al. [16] and Moreira et al. [2] These samples were obtained by jugular venous puncture using a sterile vacuum system with tubes without anticoagulant. The blood was centrifuged at 2000× *g* for 10 min, and the serum was aliquoted and stored at −35 °C for analysis.

### 2.5. Serological Analyses

The sera were analyzed for the presence of antibodies against equine brucellosis. In the diagnosis of brucellosis, the buffered acidified plate antigen (BAPA) test was used as a screening method. The BAPA test used a standardized *Brucella abortus* strain 1119/3, stained with Bengal rose, at a concentration of 8.0% cell volume and pH 3.63, and was sourced from a certified supplier compliant with the Technical Manual of the PNCEBT. The 2-ME test used a whole-cell antigen of *Brucella abortus* strain 1119/3, diluted to 4.5% in 0.85% saline solution with 0.5% phenol, also following PNCEBT standards. These antigens have been validated for equine brucellosis diagnosis in prior studies [17], ensuring specificity and sensitivity for detecting anti-*Brucella* spp. antibodies. All analyses were performed and interpreted according to the technique recommended in the Technical Manual of the National Program for the Control and Eradication of Brucellosis and Tuberculosis [7]. Positive and negative controls were included in all serological assays. Positive controls consisted of sera from equids confirmed positive for *Brucella abortus* by the 2-ME test, sourced from the Animal Health Laboratory’s reference collection. Negative controls were sera from healthy equids with no history of brucellosis exposure, as verified by prior testing. Controls were stored at −35 °C and thawed at room temperature before use to ensure stability. Testing protocols followed the PNCEBT Technical Manual, with assays performed under controlled conditions to minimize variability.

### 2.6. Epidemiological Survey

During sampling, an epidemiological questionnaire was used to gather information on animal characteristics, such as species (equine or asinine/hybrid), sex (male or female), breed (mixed-breed or purebred), age group (<2, 2–6, or >7 years), reproductive problems, type of animal (farm, sport, or carthorse), municipality/regions of origin, floodplain habitat (i.e., if the animals are moved to wetlands during the year), and cohabitation with cattle or wildlife.

### 2.7. General Characteristics of Equids

For the three types of equids (farm animal, urban carthorse, and sport horse), 1059 animals, including 1039 horses (*Equus caballus*), 13 donkeys, and 7 mules, had their samples analyzed by the BAPA screening test, then the positive samples were analyzed through the confirmatory test for brucellosis 2-ME. The samples of this study include 75 rural farms in 14 municipalities in Western Pará.

The equids used for the sport horse and the urban carthorse were not found in all municipalities. Samples of sport horses were collected in events held in the municipalities of Santarém (n = 12), Itaituba (65), Altamira (20), Rurópolis (43), and Uruará (73), as well as at the sports training center in the municipality of Brasil Novo (13). Samples of urban carthorses were collected from the following municipalities: Brasil Novo (n = 6), Itaituba (12), Óbidos (2), Santarém (31), and Terra Santa (5).

### 2.8. Statistical Analysis

Data were tabulated to calculate the frequency of seropositive and seronegative equids. Animal-level prevalence was determined as the number of positive equids divided by the total sampled, multiplied by 100, for both BAPA and 2-ME results. Herd-level prevalence considered a farm positive if at least one equid tested positive.

Univariate analysis used the Chi-square test to assess associations between *Brucella* spp. antibody prevalence and risk factors. Variables with *p* < 0.200 in univariate analysis were included in a multivariate logit binary logistic regression model with stepwise backward elimination, retaining variables with *p* < 0.05. The Hosmer–Lemeshow test evaluated model fit. A significance level of *p* < 0.05 was used for all tests, performed in Minitab 17 (Minitab Inc., State College, PA, USA). Data were analyzed using Minitab 17 software (Minitab Inc., State College, PA, USA). Odds ratios with 95% confidence intervals (95% CI) were calculated using the GraphPad Prism 6 software (GraphPad Software Inc., La Jolla, CA, USA). A significance level of 5% (*p* < 0.05) was used for all statistical tests.

## 3. Results

### 3.1. Detection of Antibodies Against Equid Brucellosis—BAPA Test

**Figure 1:**
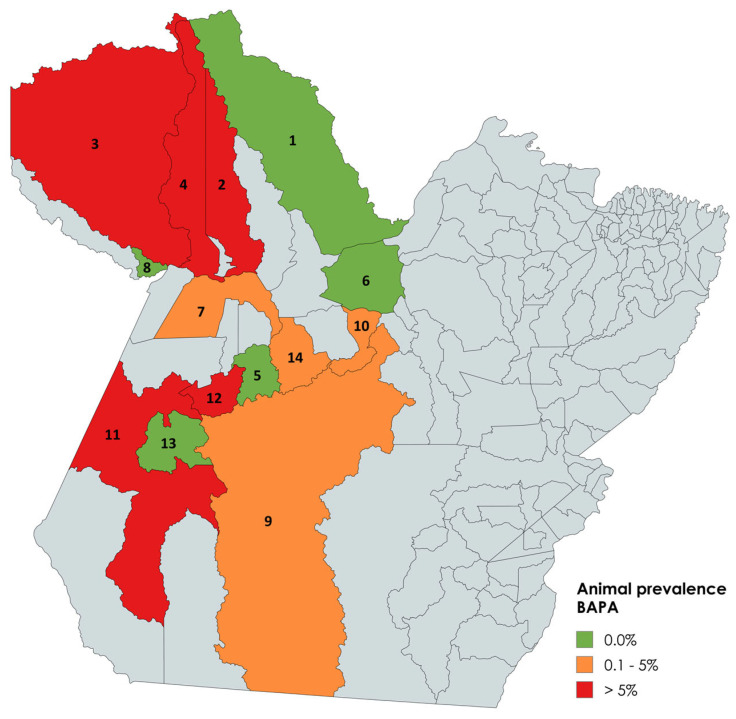
BAPA test: Prevalence of antibodies against equine brucellosis by municipality. Municipalities with 0.0% prevalence: Almeirim (1), Porto de Móz (6), Terra Santa (8), Placas (5), and Trairão (13). Municipalities with 0.1–5% prevalence: Santarém (7), Uruará (14), Brasil Novo (10), and Altamira (9). Municipalities with > 5% prevalence: Oriximiná (3), Óbidos (4), Alenquer (2), Rurópolis (12), and Itaituba (11). Gray cities were not included in the study.

Of the 1059 equids tested, 44 were reactive in the BAPA test, yielding an animal-level prevalence of 4.15% (95% CI: 3–5.5%). Positive animals were found in nine municipalities (Alenquer, Óbidos, Oriximiná, Santarém, Altamira, Brasil Novo, Itaituba, Rurópolis, and Uruará) while five (Almeirim, Placas, Porto de Móz, Terra Santa, and Trairão) had no BAPA-reactive equids. Among farm animals, 36 of 777 (4.63%, 95% CI: 3.2–6.3%) tested positive; among sport horses, 4 of 226 (1.77%, 95% CI: 0.4–4.4%); and among urban carthorses, 4 of 56 (7.14%, 95% CI: 1.9–17.3%). Figure 1 illustrates BAPA test prevalence by municipality.

Table 1 presents a risk factor analysis for the BAPA test. No differences between the prevalence of antibodies against *Brucella* spp. and animal type (*p* = 0.085), mesoregion (*p* = 0.847), microregion (*p* = 4), sex (*p* = 0.858), species (*p* = 0.200), age group (*p* = 0.526), lowland animals (*p* = 0.607), or reproductive problems (*p* = 0.374) were found. On the other hand, the risk factor breed (*p* = 0.032) was associated with the prevalence of antibodies against brucellosis, with 39 animals positive out of the 749 mixed breed equids sampled 4.95% (95% CI: 3.5–6.7) and 5 animals positive out of the 266 pure equids sampled 1.85% (95% CI: 0.6–4.2).

**Figure 2:**
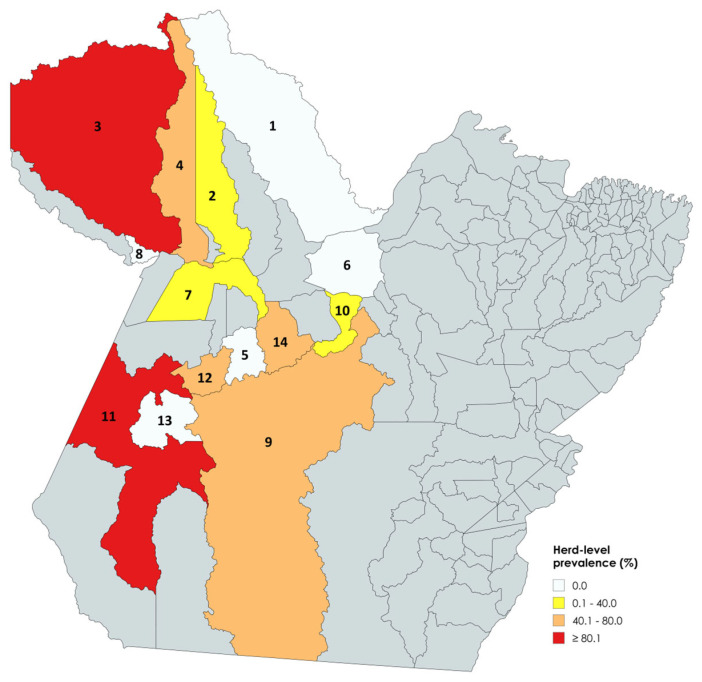
BAPA test: Herd-level prevalence of antibodies against *Brucella* spp. Municipalities with 0.0% prevalence: Almeirim (1), Placas (5), Porto de Móz (6), Trairão (13), and Terra Santa (8). Municipalities with 0.1–40.0% prevalence: Alenquer (2), Brasil Novo (10), and Santarém (7). Municipalities with 40.1–80.0% prevalence: Altamira (9), Óbidos (4), Rurópolis (12), and Uruará (14). Municipalities with ≥80.1% prevalence: Itaituba (11) and Oriximiná (3).

Herd-level prevalence (Figure 2) considering BAPA results showed farms with seropositive equids in Alenquer, Brasil Novo, and Santarém (0.1–40.0%); Altamira, Óbidos, Rurópolis, and Uruará (40.1–80.0%); and Itaituba and Oriximiná (≥80.1). Municipalities with no seropositive herds were Almeirim, Placas, Porto de Móz, Trairão, and Terra Santa.

### 3.2. Detection of Antibodies Against Brucella spp. Equid Brucellosis—2-ME Test

Of the 44 samples that tested positive for BAPA, only 11 were positive for brucellosis (2-ME) in the confirmatory test, with 18 being negative and 15 being inconclusive. This study had an animal-level prevalence of 1.03% (95% CI: 0.5–1.8) for equine brucellosis in Western Pará. Of the positive animals, 2 perform sports activities and 9 are animals raised on farms performing activities related to cattle farming. There was no positive wagon animal.

Among the 11 positive animals, all came from the Southwest Pará Mesoregion 1.41% (95% CI: 0.7–2.5), 6 from the municipality of Uruará 3.02% (95% CI: 1.1–6.5), 2 from Altamira 1.42% (95% CI: 0.1–5), 1 from Itaituba 0.55% (95% CI: 0.01–3), 1 from Brasil Novo 0.63% (95% CI: 0.01–3.4) and 1 from Rurópolis 1.05% (95% CI: 0.02–5.7).

The 15 inconclusive 2-ME results could not be retested due to insufficient serum and logistical challenges in relocating animals. The BAPA test, used for screening, is less specific than the 2-ME test, which confirms chronic infections by detecting IgG antibodies [7].

Table 2 shows the analysis of risk factors for brucellosis in horses using the confirmatory test 2-ME. There was a significant difference in the mesoregion (*p* = 0.047), where all positive animals come from the southwest of Pará. It was observed that there were no significant differences between the breed (*p* = 0.571) considering the limited number of positive animals: mixed breed 9/788—1.14% (95% CI: 0.5–2.1) and purebred 2/271—0.74% (95% CI: 0.08–2.6). Among the positive animals, there are 7 males and 4 females, 9 with aptitude for work, and 2 with reproductive activities, the latter two being of the species Equus caballus with an age of more than 7 years and without any type of reproductive problems.

**Figure 3:**
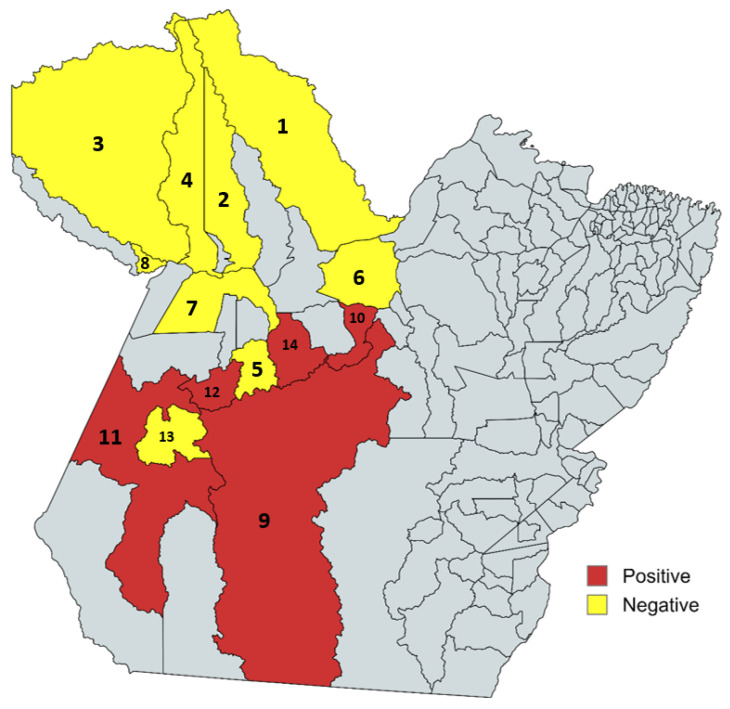
Map of positive and negative municipalities in test 2-ME. Negative municipalities: Almeirim (1), Porto de Móz (6), Alenquer (2), Óbidos (4), Oriximiná (3), Terra Santa (8), Santarém (7), Placas (5), and Trairão (13). Positive municipalities: Brasil Novo (10), Uruará (14), Altamira (9), Rurópolis (12), and Itaituba (11).

The distribution of positive and negative municipalities for antibodies against *Brucella* spp. are represented in Figure 3, with the following municipalities being negative: Almeirim, Porto de Móz, Alenquer, Óbidos, Oriximiná, Terra Santa, Santarém, Placas, and Trairão. The following municipalities are positive for brucellosis in equines from Western Pará: Brasil Novo, Uruará, Altamira, Rurópolis, and Itaituba.

**Figure 4:**
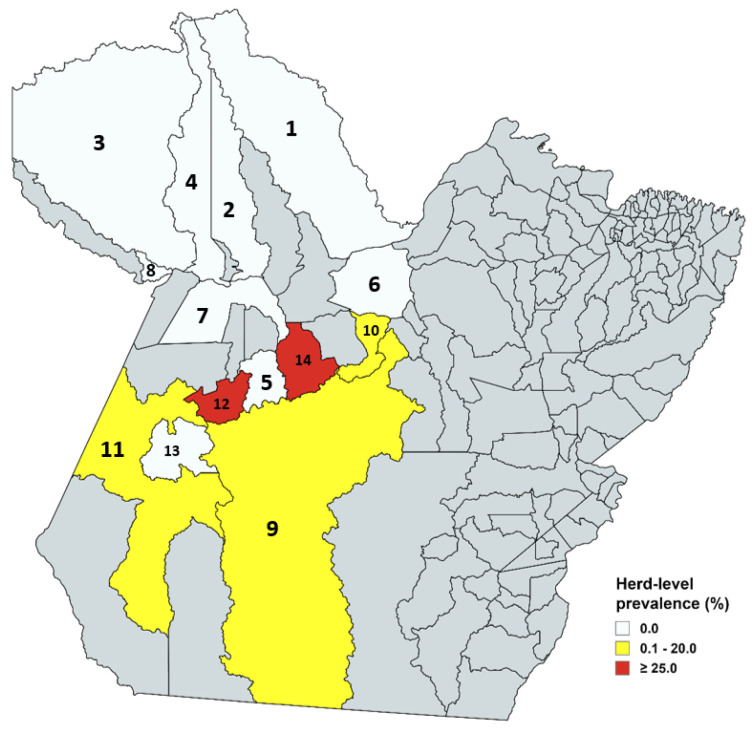
Test 2-ME: Prevalence of antibodies against equine brucellosis by herd. Municipalities with 0.0% prevalence: Almeirim (1), Porto de Móz (6), Alenquer (2), Óbidos (4), Oriximiná (3), Placas (5), Santarém (7), Trairão (13), and Terra Santa (8). Municipalities with 0.1–20.0% prevalence: Altamira (9), Brasil Novo (10), and Itaituba (11). Municipalities with ≥25.0% prevalence: Rurópolis (12) and Uruará (14).

Regarding the prevalence of brucellosis by farm/herd through the 2-ME test, it can be observed that of the 75 farms sampled, in 8 there was at least one seropositive animal, 10.66%. The herd-level prevalence can be seen in Figure 4, which characterizes the municipalities that obtained negative herds—Almeirim, Porto de Móz, Alenquer, Óbidos, Oriximiná, Terra Santa, Santarém, Placas, and Trairão—and municipalities with positive herds—Brasil Novo, Uruará, Altamira, Rurópolis, and Itaituba.

## 4. Discussion

This study assessed the prevalence of *Brucella* spp. antibodies in farm animals, urban carthorses, and sport horses in Western Pará. Due to regional diversity, not all equid types were present in every municipality, with sport horses sampled in six municipalities and carthorses in five. The BAPA screening test indicated a seroprevalence of 4.05% (95% CI: 3.0–5.5%), while the 2-ME confirmatory test confirmed a prevalence of 1.03% (95% CI: 0.5–1.8%), which align with the global prevalence of the disease 1.92% [8]. This is consistent with results found by Resende et al. [12] in Soure, Marajó Island, Pará, and other Brazilian studies in Rio Grande do Norte, Northeast region [5], and Minas Gerais, Southeast region [17]. Similar low prevalence was seen in “baixadeiro” horses (a small-sized Brazilian breed) from the lowlands of Maranhão [18] and urban carthorses from Paraná [19]. Higher prevalence’s (6.5%) were also reported in the semiarid region of Northeast Brazil [20], with frequency of positive animals as high as 73% in horses with clinical symptoms of bursitis [21].

Equine brucellosis poses a significant *One Health* challenge due to its zoonotic potential and ecological implications. The close contact between equids, humans, and other livestock in Western Pará underscores the need for integrated surveillance. Recent advances in proteomics-based diagnostics [22,23] offer promising tools for improving diagnostic specificity and supporting One Health frameworks for brucellosis control.

No significant associations were found between seropositivity and animal type, sex, species, age, or reproductive problems. The significant association with breeds in the BAPA test (*p* = 0.032) was not confirmed by 2-ME (*p* = 0.571), suggesting false positives in screening. Previous reports [5,17] also did not reveal the existence of an association between variable sex and age and seropositivity of *Brucella* spp. The low number of positive samples (11) in the 2-ME confirmatory test and the presence of zero counts in some categories (e.g., carthorses, asinine/hybrid) limited the statistical power of certain risk factor analyses. This is a common challenge in low-prevalence settings, where small sample sizes in specific subgroups can lead to invalid Chi-square approximations [14]. Future studies with larger sample sizes or targeted sampling in high-risk areas could improve the robustness of these analyses. It is noteworthy the absence of seropositive animals among mules, which may reflect their low sample size (n = 7).

False positive results in the BAPA test may occur due to cross-reaction with other gram-negative bacteria that have a bacterial wall constitution similar to the genus *Brucella* [24]. The 2-ME test, detecting IgG antibodies, confirms chronic infections with greater specificity but may miss early infections [3,25,26,27]. Interpretations of test results according to the PNCEBT should also be considered [7].

The serum samples used in this study, sourced from Minervino et al. and Moreira et al., were stored at −35 °C, a standard condition for preserving antibody stability in serological assays. The samples were analyzed using the BAPA and 2-ME tests. The BAPA test, primarily detecting IgM antibodies, may overestimate prevalence due to cross-reactivity with other gram-negative bacteria, leading to potential false positives. In contrast, the 2-ME test, which targets more stable IgG antibodies, offers greater specificity for *Brucella* spp., confirming lower prevalence. However, its sensitivity may be limited for early infections. Emerging proteomics-based diagnostics [22,23] could improve surveillance by identifying specific *Brucella* biomarkers, reducing false positives and enhancing detection in low-prevalence settings.

A limitation of this study was the inability to retest 15 inconclusive 2-ME samples due to insufficient serum and logistical constraints in relocating animals, a common challenge in rural epidemiological studies. Future research could employ sample biobanking to preserve sera for additional testing or implement point-of-care diagnostics, such as lateral flow assays, to enable real-time confirmatory testing in remote regions.

Considering only the farm horses, 8 out 75 farms (10.66%) had at least one equid 2-ME seropositive. Even with the low animal prevalence observed, there is a high dispersion of the disease in the western region of Pará. Cohabitation with cattle, a hypothesized risk factor, was not statistically significant (*p* > 0.05) due to the high proportion of farm equids exposed to cattle and the low number of confirmed positives (n = 11). This limited statistical power for some risk factors, a common issue in low-prevalence settings. Although the transmission mechanism of equine brucellosis is not well understood, it is considered that the infection is favored by cohabitation with other domestic species. It is suggested that transmission occurs through the ingestion of water and food contaminated by vaginal discharges, abortion, and placenta remains, especially from cattle [3,10]. Cattle brucellosis in Pará ranged from 3 to 10% [28,29] in early reports, but cattle carcasses condemned due to brucellosis during slaughtering was 0.1% [30]. The Southwest Pará Mesoregion showed higher prevalence (*p* = 0.047) of *Brucella* spp. antibodies in equids, possibly due to regional differences in cattle cohabitation or management practices, similar to the cattle results observed by Roma et al. [30], where all positive animals occurred in the southwest region.

In Brazil, epidemiological data on equine brucellosis are scarce and few studies have been developed in horses, making it difficult to observe the distribution of the disease nationally [9,10,11]. Equine brucellosis poses a One Health challenge due to its zoonotic potential. The PNCEBT’s focus on bovine brucellosis limits equine-specific control measures, emphasizing the need for targeted surveillance [7].

Thus, the results of the present study confirm the potential risk of the equine species to public health, with this being the first report of horses seropositive for brucellosis in Western Pará, which contributes to the knowledge of the epidemiology of the disease.

## 5. Conclusions

This study confirms the presence of antibodies against *Brucella* spp. in equids in Western Pará for the first time, with a prevalence of 1.03% (95% CI: 0.5–1.8) based on the 2-ME confirmatory test. The higher prevalence in the Southwest Pará Mesoregion and the presence of positive animals on 5 of 14 municipalities and 8 of 75 sampled farms highlight the need for enhanced surveillance and control measures to mitigate the zoonotic risk of equine brucellosis in the region.

## Figures and Tables

**Table 1:** Risk factors for the animal-level prevalence of antibodies against *Brucella* spp. in equids using the BAPA test in Western Pará, Brazil.

Risk Factor	Positive/Total	Prevalence (95% CI)	*p*-Value	OR (95% CI)
*Type of animal*			0.085	^β^
Carthorse	4/56	7.14% (1.9–17.3)		
Sport	4/226	1.77% (0.4–4.4)		
Farm	36/777	4.63% (3.2–6.3)		
*Mesoregion*			0.847	^β^
Lower Amazon	11/278	3.96% (1.9–6.9)		
Southwest Pará	33/781	4.23% (2.9–5.9)		
*Microregion*			4	^β^
Almeirim	0/15	0.00%		
Altamira	13/499	2.61% (1.3–4.4)		
Itaituba	20/282	7.09% (4.3–10.7)		
Óbidos	6/90	6.67% (2.4–13.9)		
Santarém	5/173	2.89% (0.9–6.6)		
*Sex*			0.858	^β^
Female	18/447	4.03% (2.4–6.2)		
Male	26/612	4.15% (2.7–6.1)		
*Species*			0.200	^β^
Equine	42/1039	4.04% (2.9–5.4)		
Asinine/hybrid	2/20	10% (1.2–31.6)		
*Breed*			0.032	2.77 (1.1–7.1)
Purebred	5/271	1.85% (0.6–4.2)		
Mixed breed	39/788	4.95% (3.5–6.7)		
*Age group*			0.526	^β^
<2	2/39	5.13% (0.6–17.3)		
2 to 6	13/398	3.27% (1.7–5.5)		
>7	29/622	4.66% (3.1–6.6)		
*Floodplain habitat*			0.607	^β^
No	38/940	4.04% (2.9–5.5)		
Yes	6/119	5.04% (1.9–10.6)		
*Reproductive problems*			0.374	^β^
No	43/1048	4.10% (2.9–5.4)		
Yes	1/11	9.09% (0.2–41.20		

^β^ Odds ratio is not available because the variable was not maintained in the final model of the multivariate analysis. OR: odds ratio.

**Table 2:** Risk factors for the prevalence of antibodies against brucellosis in equids with confirmatory 2-ME test in Western Pará.

Risk Factor	Positive/Total	Prevalence (95% CI)	*p*-Value
*Type of animal*			^α^
Carthorse	0/56	0.00%	
Sport	2/226	0.88% (0.1–3.1)	
Farm	9/777	1.16% (0.5–2.9)	
*Mesoregion*			0.047
Lower Amazon	0/278	0.00%	
Southwest Pará	11/781	1.41% (0.7–2.5)	
*Microregion*			^α^
Almeirim	0/15	0.00%	
Altamira	9/499	1.80% (0.8–3.3)	
Itaituba	2/282	0.71% (0.08–2.5)	
Óbidos	0/90	0.00%	
Santarém	0/173	0.00%	
*Sex*			0.693
Female	4/447	0.89% (0.2–2.2)	
Male	7/612	1.14% (0.4–2.3)	
*Species*			^α^
Equine	11/1039	1.06% (0.5–1.9)	
Asinine/hybrid	0/20	0.00%	
*Breed*			0.571
Purebred	2/271	0.74% (0.08–2.6)	
Mixed breed	9/788	1.14% (0.5–2.1)	
*Age group*			^α^
<2	0/39	0.00%	
2 to 6	1/398	0.25% (0.006–1.3)	
>7	10/622	1.61% (0.7–2.9)	
*Use*			^α^
Sport	0/19	0.00%	
Reproduction	2/299	0.67% (0.08–2.4)	
Work	9/741	1.21% (0.5–2.3)	
*Reproductive problems*			^α^
No	11/1048	1.05% (0.5–1.8)	
Yes	0/11	0.00%	

^α^ Cells with expected counts less than 1. Cells with expected counts less than 5. Invalid Chi-Square approximation probability.

## Data Availability

The raw data supporting the conclusions of this article will be made available by the authors on request.
